# Demographic Costs Associated with Differences in Habitat Space Occupancy

**DOI:** 10.1371/journal.pone.0165472

**Published:** 2016-11-16

**Authors:** Kelly A. Williams, Donald B. Miles

**Affiliations:** Department of Biological Sciences, Ohio University, Athens, OH 45701, United States of America; Charles University, CZECH REPUBLIC

## Abstract

Delimiting the habitat characteristics describing the environmental conditions required by a species has become a critical tool for predicting organismal responses to environmental change. Grinnell emphasized the effects of environmental factors on the ability of a population to maintain a positive growth rate, yet few studies have included demographic or reproductive data in analyses of the Grinnellian niche. Identifying differences in habitat exploitation patterns in response to structural variation in the environment presents an incomplete description of the ability of species to adapt to changing habitats if demographic traits are not included. We estimated the vegetation characteristics used by individuals within a population of hooded warblers (*Setophaga citrina*) across a spatial transect that includes three structurally different forest habitats. We predicted individuals should select similar structural characteristics within each habitat and have similar reproductive success across sample sites. In the two years post burn, adults were present but no young fledged indicating the habitat requirements necessary for reproduction were absent in this habitat. We found significant differences in habitat space occupied by individuals in unaltered and harvested habitats. Nesting habitats used by female warblers differed from available habitat. Fledging success was lower in the harvested habitat 10 to 12 years post-harvest. In the harvested habitat, fledging success was greater on mesic slopes but decreased along a habitat gradient to xeric ridgetops, suggesting compensation in habitat use does not ameliorate fitness costs. In contrast, there was no difference in the number of fledged young along this gradient in the unaltered habitat. Based solely on occupancy data, traditional ecological niche models would incorrectly conclude the environmental characteristics found across the three forested habitats are included in the Grinnellian niche of the hooded warbler. However, examination of demographic and environmental data simultaneously allows differentiation between occupied habitat space and niche space.

## Introduction

The distribution of species across a landscape depends on the spectrum of both biotic and abiotic factors that result in a stable or positive population growth rate [1–3]. The combination of these factors constitute a species’ niche [1,2,4,5]. The habitat requirements that restrict a species to a geographic range where there are the necessary environmental factors for survival and reproduction are components of the Grinnellian niche [6,7]. These environmental factors include ambient temperature, precipitation and habitat structure that ultimately affect the availability and distribution of resources, which in turn determines the suitability of a habitat for a species and delimits its range [8–11]. This niche space consists of multiple habitat axes because different environmental characteristics may be needed for different aspects of a species’ life history [2,12]. Some habitats may contain resources along critical axes, such as for foraging, but insufficient resources for other aspects of reproductive success, such as nesting sites, and thus be a sink habitat [13].

A common goal of niche mapping studies is to link habitat variables with estimates of abundance, density or species richness [7,14]. Ecological niche modeling is a correlational method for determining the environmental conditions associated with a species’ distribution or abundance [9–11]. The distributional patterns are based on broad scale occupancy and estimates of environmental factors at the macroecological level. However, these patterns can be associated with spatially correlated landscape features and biased due to mode of sampling [10,15]. For example, the United States Geological Survey (USGS) North American Breeding Bird Survey (BBS) data are collected along roads yet species habitat associations along edges may differ from core habitats [16,17]. Therefore, edge habitats may not represent the center of the environmental space required for stable or positive population growth and instead be indicative of suboptimal habitat occupied by floaters or individuals that cannot obtain territories in higher quality habitat [18].

A key aspect of the Grinellian niche is that a species range coincides with those environmental conditions where population growth rate is stable or positive [4]. Despite attempts to clarify terminology when discussing the niche [4,19,20], confusion in usage still exists. Here we make two distinctions. First, “habitat space” includes the habitat characteristics that permit the presence of a species. This habitat space represents occupancy of the species but may include space occupied by sink, as well as, source populations. Second, we define the Grinnellian niche as the subset of non-consumable resources that a species occupies that are tied to estimates of fitness which suggest the species is able to maintain stable or positive population growth. Relatively few studies integrate demographic data in the analysis of niche variation or have elucidated the combination of structural habitat characteristics that predict successful reproduction and survival. Including demographic data enhances the understanding of how fine-scale environmental conditions affect variation in source sink dynamics across the landscape and can help to better differentiate between habitat space that permits occupancy and habitat space that supports source populations.

Multivariate techniques can be used to identify habitat characteristics along different axes. Variation in the habitat characteristics used by individuals, populations or species may be evaluated using the axes describing a habitat space [12,21,22]. The habitat space used by individuals within and among populations can be compared. Thus, we can determine whether the habitat space along different axes expands, shrinks or differs in position or shape across structurally different habitats [23]. Including data on reproductive success or survival allows the ability to establish a connection between the habitat characteristics and source sink dynamics. Habitat space that supports a population growth of ≥ 0 would lie within a species niche space. Less productive and sink habitats can be identified by a difference in position in habitat space that is also associated with a reduction in reproductive success compared to either prior conditions or another habitat.

Here, we evaluate the habitat space occupied by a population of breeding hooded warblers (*Setophaga citrina*) among structurally different forest habitats. Because many forests exhibit variation in disturbance, we chose forest sites with altered structural features in different strata (e.g., understory and subcanopy) to determine the relationship among habitat structure and reproductive success. Because adult hooded warblers were observed in all three (unaltered, harvested and burned) habitats during the breeding season, we predicted that individual warblers should track components of their Grinnellian niche across the habitat gradient.

### Natural history of hooded warblers

Hooded warblers are a Neotropical migrant species that inhabit mature deciduous forests across the Eastern United States during the breeding season. These forests are typically dominated by *Quercus* spp. (oak), *Carya* spp. (hickory), *Acer* spp. (maple) and *Fagus* spp. (beech) and have a well-developed understory and shrub layer for nesting [24]. Despite considerable variation in plant species composition across the breeding range, there are similarities in the habitat structure associated with the forested habitats occupied by hooded warblers. These structural similarities include forests with a high tree species diversity in the canopy and subcanopy and high density of stems in the shrub layer compared to more even aged forests with a high coverage of large saplings and understory trees [25].

In many species, males and females are known to require sex-specific habitat characteristics [26–28]. For example, when there are differences between sexes in behavior contexts such as foraging [29]; males and females may use different cues to select habitat. Male hooded warblers that have been captive reared preferentially orient toward vertical structure while captive reared females orient toward oblique structure [30]. Furthermore, male and female hooded warblers use structurally different habitat during the winter [26,31]. During the breeding season, hooded warbler males sing in the midstory and favor territories with greater vertical structure (e.g., mature forest with an open midstory) to enhance territorial defense and their ability to attract mates. In contrast, females use habitats with increased understory structure (e.g., complex, oblique structure like in brier thickets) for nest concealment in the shrub layer and do not exclusively use their social partner’s territory during the breeding season [32]. Although nest concealment was not associated with nest survival [33], high understory coverage at the scale of the territory may increase foraging opportunities for the female and fledged young and decrease predation because predators have more substrate to search.

#### Study objectives

We quantified the habitat space of males (territory centered habitat characteristics) and females (nest site habitat characteristics) during the breeding season in three structurally different forest habitats within an 80–100 yr old second growth forest. One habitat was undisturbed, a second was subjected to prescribed burns, and a third forest habitat that was subjected to a selective harvest by mechanical thinning. We used this variation to determine whether individuals selected similar features across sites. Moreover, we investigated whether there were differences in habitat space use between the sexes. We hypothesize that male and female hooded warblers should exploit different environmental characteristics within a habitat contingent on behavioral contexts (e.g., foraging, reproduction). We predicted each sex requires a similar habitat space along the foraging axis, but require different habitat characteristics along the territorial defense (male) and nesting (female) niche axes. We also had specific predictions for each forest habitat based on the type of disturbance. Fire results in the near complete elimination of the shrub layer, which reduces the habitat space available for foraging and nest placement. Thus, we predicted that males would sing and defend territories in the burned habitat but that reproductive success would be low as a consequence of unfavorable understory structure required for nest placement. Overall, we predicted a reduction of the Grinnelian niche space in burned habitats. Mechanical thinning entails removing a fraction of the basal area of a forest to increase the amount of sunlight that penetrates the canopy and reaches the forest floor. Thinned forests have increased growth in the shrub and understory layers which results in a more heterogeneous and structurally complex understory. Hooded warblers are associated with canopy gaps [34,35] and often invade selectively logged forests 1–5 years post logging [35–37]. Therefore, we predicted that there would be increased reproductive success in the harvested habitat. In addition, we predicted that the habitat space would be larger and the position in habitat space would include more complex understory vegetation in the harvested habitat compared to undisturbed habitat. Finally, we determined if reproductive success, as estimated by the number of fledged young per nest, is related to habitat structure. We predicted that hooded warbler nest success would be positively correlated with complex understory vegetation structure.

## Materials and Methods

### Study site

We monitored habitat use and reproductive success of hooded warblers at Tar Hollow State Forest (39°33’0”N, 82°76’7”W), which is located in southern Ohio within the unglaciated Allegheny Plateau. The primary canopy species include *Quercus alba* (white oak), *Quercus prinus* (chestnut oak), *Carya* spp.(hickories), and *Quercus rubus* and *Quercus velutina* (red and black oak; [38]). Understory vegetation includes *Smilax* spp. (greenbrier), *Viburnum acerifolium* (maple-leaf viburnum), *Rubus* spp., *Lindera benzoin* (spicebush) and many small-diameter saplings (i.e., < 10 cm DBH [diameter at breast height]). We established study sites (20–30 ha) at three locations within the forest. We selected sites with divergent physical structure that mimicked different stages of disturbance, yet attempted to control for other environmental characteristics (e.g., moisture, slope, elevation). One site had experienced no logging for the past 80–100 years (unaltered habitat). Another site had been thinned from below in 2001 and resulted in an approximately 25% reduction in basal area (harvested habitat). The selective harvest focused on removing midstory and subcanopy trees [39]. This manipulation created canopy gaps, but did not change overall canopy height and the density of canopy trees (>38 cm DBH). The main effect of the disturbance was to alter the understory vegetation density and species composition [40]. For example, there was an increase in early-successional saplings such as *Liriodenron tulipifera* (tulip tree) and *Sassafrass albidum* (sassafras) and an increase in *Rubus* spp. The third site (burned habitat) had been burned during the winter or early spring prior to leaf-out in 2001, 2005, and 2010. This study site had reduced understory vegetation structure and low shrub cover [40,41] in the years immediately following burning (e.g., 2010, 2011).

### Ethics statement

This study and all protocols to collect data for this research were approved by the Ohio University Institutional Animal Care and Use Committee (permit H12-02). In addition, all bird banding and related methods were conducted as permitted under USGS banding permit #23434.

### Capture methods

We captured hooded warblers using mist nets during late April through mid July in 2010 and 2011. Each captured bird was banded with a USGS numbered band and a unique combination of color bands for individual identification in the field. We also individually marked and weighed nestlings when they were between four to six days of age.

### Territory mapping, nest searching and monitoring

We engaged in an exhaustive search for nests and territorial birds starting in early May through mid-July during the 2010 and 2011 breeding seasons. We varied the direction and starting location on each visit to ensure that locations within the forest were sampled at different times of day throughout the breeding season. We georeferenced all hooded warbler observations and nest locations. When observed, we identified individuals by their unique color band combinations. Male territories were defined by the detection of an individual in a location on at least three survey days spanning a period greater than 10 days apart. We also used behavioral interactions (e.g., territorial defense) with neighboring males following Bibby et al. [42]. We monitored nests every one to three days following the Breeding Bird Research and Monitoring Database program (BBIRD) protocol [43] to determine nest fate.

### Habitat assessment

We established and measured habitat characteristics in 104 habitat plots (11.3 m radius circle = 0.04 ha each) that portrayed different types of use by hooded warblers following the BBIRD protocol [43]. We described the structure and composition of habitat available to warblers by selecting 10 random plots (“random plot”) in each forest habitat, 100 m apart (using a random number generator). We also measured vegetation characteristics in habitat plots used by hooded warblers: habitat plots were established in the center of each male's territory (“territory plot”) and centered on each nest (“nest plot”) after breeding was completed. Only nine random plots were retained for analysis in the harvested habitat because one territory overlapped with a random plot. The smaller size (~20 ha) of the burn site prevented us from establishing more than five random habitat plots that were 100 m apart. We modified the BBIRD protocol slightly (change in size class assignment, below) in our estimation of canopy, shrub, and sapling cover. We recorded trees by species and size class (10–23 cm, 23–38 cm, > 38 cm DBH) in the 11.3 m radius vegetation plots. We recorded all shrubs and saplings in a 5 m radius plot (centered within the 11.3 m radius plot) by species. We recorded the number of vertical stems at 10 cm above the ground by species and assigned to three size classes (< 3 cm, 3–6 cm, and 6–10 cm).

### Statistical analyses

Because of small sample sizes for some woody plant species and size classes, we excluded all plant species with fewer than five occurrences in the data (combined random, nest and territory habitat plots). These included stems in the medium and large shrub size classes (3–6 and 6–9 cm). In addition, we grouped *Quercus* spp. as white oak (*Q*. *alba*, *Quercus muehlenbergii*), and red oak (*Q*. *rubra*, *Q*. *velutina*). Although *Q*. *prinus* (chestnut oak) is a white oak, it was recorded separately because of the high abundance of this species as well as differences in habitat characteristics (e.g., plant species composition, structure) in areas where chestnut oak occurs (e.g., xeric ridge tops) compared to other white oaks. We grouped all *Carya* spp. (hickory) by genus and we grouped different species of *Smilax* and *Rubus* by genus (*Smilax* spp. and *Rubus* spp.). We conducted all analyses in R [44].

#### Defining a habitat space

We used nonmetric multidimensional scaling (NMDS), an ordination technique that compares the relationships among individual objects (e.g., habitat plots) in a multidimensional space [45]. The distance between objects in a multidimensional space reflects their similarity or dissimilarity. In our analysis, the volume occupied by the objects is an estimate of the habitat space used by individuals. The goal of this analysis was to compare the similarity in habitat space occupied among individuals in the three habitats and between males and females in relation to the availability of habitat.

We applied a square-root transformation to better normalize count data and Wisconsin double standardization (species divided by maxima and sites standardized to equal totals) to visually examine the habitat axes within the forest. We used three dissimilarity metrics in vegan [46]. 1. Bray-Curtis index,

$$d_{jk}=\frac{\sum_{i}{|x}_{ij}-x_{ik}|}{\sum_{i}\left|x_{ij}+x_{ik}\right|}$$

where x_ij_ and x_ik_ are the count of species for each site (j and k).

2. Jaccard index,

$$d_{jk}=\frac{2B}{\left(1+B\right)}$$

where B is Bray-Curtis dissimilarity.

3. Kulczyński index,

$$d_{jk}=1-0.5(\frac{\sum_{i}\min\left(x_{ij},x_{ik}\right)}{\sum_{i}x_{ij}}+\frac{\sum_{i}\min\left(x_{ij},x_{ik}\right)}{\sum_{i}x_{ik}})$$

which considers the minimum count of species at each site. The Kulczyński dissimilarity coefficient is robust to situations where sites have very common or rare species [45]. We compared the performance of the three measures using the function rankindex in package vegan [46] to determine which dissimilarity index best separated the habitat community characteristics in relation to a gradient in canopy cover. All three indices were found to perform similarly. We used Jaccard dissimilarity coefficient because it is more effective in preserving the relationships among objects (i.e., habitat plots) in the multidimensional space [46,47]. The goal of ordination analyses is to summarize a complex multidimensional dataset in a fewer number of axes. In order to determine the number of axes to retain for interpretation, we compared the goodness of fit for two, three and four axes solutions. We compared the stress for each solution, which is defined as the difference between the original distance between objects and their position in the reduced space. We used the NMDS solution that resulted in the lowest stress.

To assist in visualization and interpretation of the relationships among the habitat characteristics associated with male and female habitat use, we plotted the 95% confidence ellipses based on the standard deviation of the weighted averages from the centroid (function ordiellipse), as well as, the convex polygon (function ordihull) for each plot type. We interpreted the axes describing the habitat space by calculating the correlations between the habitat characteristics and the NMDS axes. The method is similar to interpretation of Principal Components Analysis (PCA) factor loadings. We used the vegetation variables with the highest correlation coefficients to interpret each axis. Because individuals are plotted in this space based on the habitat characteristics they use, we can determine the habitat characteristics that are components of the habitat space occupied by individuals.

#### Is there a difference in the position in habitat space?

We determined the similarity in vegetational composition and structure among the three forest types and the overlap in habitat space in males and females by plotting 95% confidence interval (CI) ellipses of the centroids by forest habitat type and sex using the function ordiellipse in the package vegan. The CI ellipses allow visual comparison of overlap in habitat space among different groups. We also tested for differences in habitat space among habitats and by sex with function adonis in the package vegan [46,47]. Adonis is a non-parametric permutational multivariate analysis of variance (PERMANOVA) based on dissimilarities that tests the hypothesis of no difference in the centroids within a multivariate space [23]. We used Jaccard dissimilarity coefficient and 5,000 permutations with habitat type (unaltered, harvested, burn) and used plot type (random, female nest, male territory) as predictor variables to test the hypotheses that there were no differences in habitat space occupied among the male territory, female nest, and random plots or by habitat type. Both the visual comparison of the 95% confidence ellipses and PERMANOVA provide tests to determine whether variation in niche space remains similar across habitats. Alternatively, birds may alter their use of habitat characteristics depending on the environmental context due either to the availability of non-consumable resources or the interaction between available environmental and biotic conditions. In addition, visual inspection of size and orientation of the confidence ellipses allow us to determine if the centroids of the occupied habitat space differs among environments and if this space expands or shrinks for different groups. Thus, these analyses allow us to determine the habitat characteristics associated with any observed differences in habitat space occupancy.

We only observed three males whose territories were completely within the burned habitat (other males were present but territories encompassed areas in adjacent undisturbed habitat) and two nests during the two years of this study so all habitat plots from the burn were removed from further analyses and we only made comparisons of habitat space for male territory and female nest sites in the unaltered and the selective harvest habitat. We used function adonis with 5,000 permutations to determine if there were differences in the habitat characteristics used for male territories and for female nest locations in the unaltered compared to the harvested habitat and if there were differences in the habitat space by sex in separate analyses. We also used function adonis to test for differences in habitat characteristics between random and territory plots and random and nest plots.

#### Is there evidence of a reduction or expansion of habitat space?

We used homogeneity of dispersions test (function betadisper in vegan), a multivariate analogue to Levene's test of equality of variances, to compare the median distances from the centroid of the male territory and female nest site characteristics in each habitat to determine if there were differences in the dispersion (increase or decrease in the volume of habitat space). The homogeneity of dispersion test allows us to determine if the habitat space of different groups (e.g., individuals breeding in structurally different habitats, or by sex) remains the same, shrinks or expands. Pairwise differences in dispersion among groups were compared using Tukey's HSD test with 999 permutations. Significant increases (or decreases) in dispersion between groups indicate an increase (or decrease) in habitat space. We used homogeneity of dispersion with 1,000 permutations to determine if the dispersion in habitat space (all habitat plots combined) differed between the unaltered compared to the harvested habitat. We then used separate homogeneity of dispersion tests with 1,000 permutations to determine if there were differences in the size of the habitat space for male territory and female nest site characteristics by forest habitat.

#### The Grinnellian niche: Are differences in habitat space related to reproductive success?

We tested the hypothesis that forest habitat and the structural habitat characteristics explained variance in reproductive success using a generalized linear model with a Poisson distribution. We used the number of fledged young per nest as our estimate of reproductive success and the scores from the first two NMDS axes as our predictor variables. We used function glmulti [48] in R to select the most supported of all possible candidate models from the full model with all interactions (forest habitat x NMDS1 x NMDS2) using the small sample corrected AICc [49].

## Results

**Fig 1:**
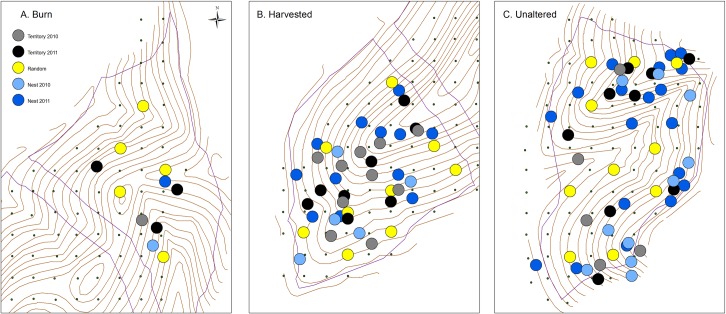
Map illustrating the location of the habitat plots used to estimate vegetation composition and structure. We show the positions for the different plot types (male territory centered, nest centered, and random) each year in (A) burn habitat, (B) harvested habitat and (C) unaltered habitat.

We obtained data on the structural characteristics of the vegetation in 53 habitat plots (Fig 1) in the unaltered habitat (*n* = 10 random plots, *n* = 14 territory centered plots, *n* = 29 nest centered plots), 41 plots in the selective harvest (*n* = 9 random plots, *n* = 14 territory centered plots, *n* = 18 nest centered plots), and 10 plots in the burn habitat (*n* = 5 random plots, *n* = 3 territory centered plots, *n* = 2 nest centered plots). The best NMDS solution had four axes and a stress of 15.99%. A two axis solution had a stress value of 28.3%. The first NMDS axis described a moisture gradient with mesic forest characterized by *L*. *benzoin* and canopy *Liriodendron tulipifera* at one end to xeric forests associated with canopy *Q*. *prinus*, *Smilax* spp., *Q*. *prinus* saplings and midstory *Acer rubrum* at the other (S1 Table and S1 Fig). Canopy and subcanopy *Q*. *prinus* were positively correlated with the second axis indicating increased complexity in the canopy. In contrast, the second axis was negatively correlated with canopy *Q*. *alba*, and *Q*. *rubra* and *Q*. *alba* saplings indicating increased understory complexity. Thus, the second axis described a structural gradient from increased complexity in the understory to increased complexity in the canopy.

### Is there a difference in the position in habitat space?

**Fig 2:**
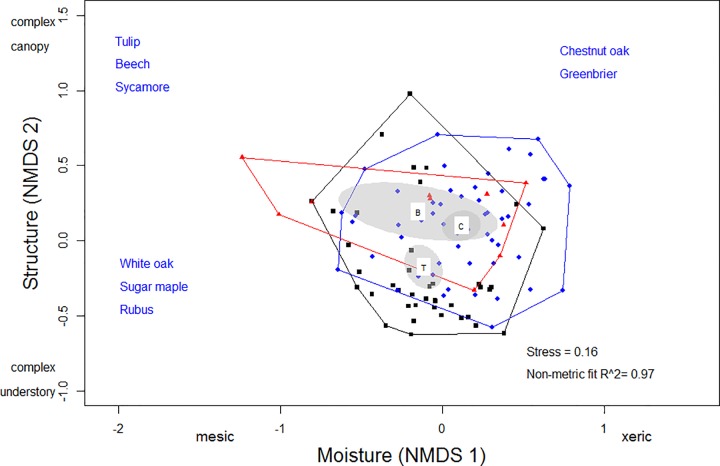
Charcaterization of habitat space in the unaltered, burned and harvested habitat. Results of a nonmetric multidimensional scaling analysis of similarity in the vegetational composition and structure in random habitat plots, nest habitat plots and male territory habitat plots from the unaltered (circles), selective harvest (squares) and the burn habitat (triangles). Convex hulls are plotted for each stand and 95% confidence ellipses based on the standard deviation of the weighted distance from the group centroid are indicated (C = unaltered habitat, B = burned habitat, T = selective harvest habitat).

We found a significant difference in the centroids of the habitat space among the unaltered, burn and selective harvest habitats (PERMANOVA: *F*_*2*,*95*_ *=* 4.5, *P =* 0.002); however, output from function adonis does not indicate which centroids differ when more than two groups are compared. Inspection of the 95% confidence ellipses from the NMDS (Fig 2) suggest that the habitat characteristics in the burn and unaltered habitats were more similar to each other (centroids overlapped), but differed from the harvested habitat. A similar pattern was observed when comparing only the random habitat plots in this habitat space (S2 Fig). In addition, male territory, female nest and random habitat space differed (PERMANOVA: *F*_*2*,*95*_ *=* 1.5, *P* = 0.02). We found no significant interaction among male, female and random habitat characteristics by forest habitat (PERMANOVA: *F*_*4*,*95*_ *=* 0.85, *P* = 0.84).

**Fig 3:**
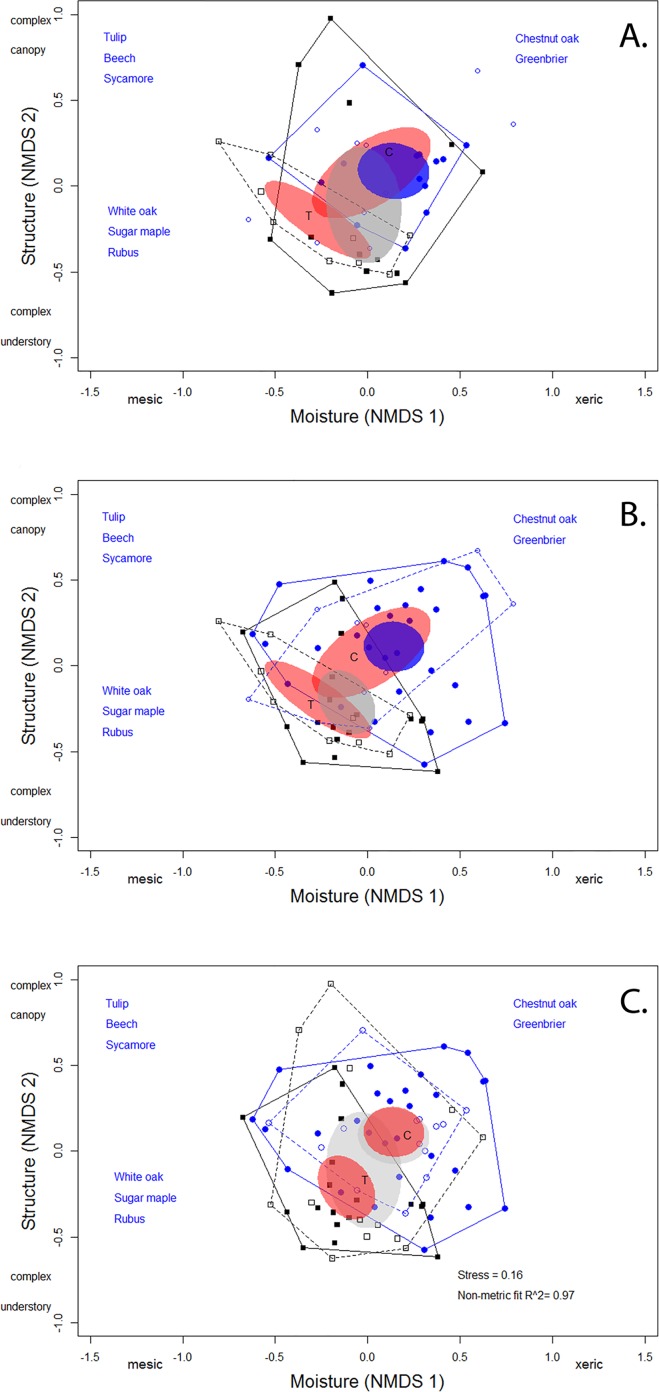
**Comparison of habitat space between (A) male territories and random space, (B) female nest sites and random space, and (C) male territories and female nest sites.** The position of the habitat plots in a space defined by the first two axes from the NMDS ordination in the unaltered (circles), and selective harvest (squares). Solid symbols represent territory plots (A) or nest plots (B) while open symbols represent random plots. Convex hulls define habitat space in each habitat (solid line = Territory; dashed lines = Random, blue = unaltered habitat and black = harvested habitat) and 95% confidence ellipses based on the standard deviation of the weighted distance from the group centroid (territories or nests in unaltered habitat = blue oval, territories or nests in harvested habitat = gray oval, random plots = red ovals). (C) Solid shapes represent nests sites and open shapes represent male territories. Convex hulls are drawn for female and male habitat space in each habitat (solid line = female nest; dashed lines = male territory) and 95% confidence ellipses based on the standard deviation of the weighted distance from the group centroid are indicated (female nest sites = red ovals; male territory = gray ovals).

We compared the random and territory habitat space in the unaltered and harvested habitats to determine if males were selecting components of the available (random) habitat. While the habitat space in the two habitats overlapped (Fig 3A), we found a significant difference between the centroids of the habitat characteristics in the unaltered compared to the harvested habitat (PERMANOVA: *F*_1,46_ *=* 2.65, *P* < 0.001). We did not find a difference between random habitat plots and male territory characteristics overall (PERMANOVA: *F*_1,46_ *=* 1.25, *P* = 0.18; Fig 3A) and the interaction between habitat type and plot type was not significant (PERMANOVA: *F*_1,46_ *=* 0.81, *P* = 0.71).

We compared the random and nest habitat space in the unaltered and harvested habitats to determine if females were selecting nest sites that differed from the available habitat (Fig 3B). We found a significant difference between the centroids of the habitat characteristics used for nesting in the unaltered compared to the harvested habitat (PERMANOVA: *F*_1,65_ *=* 4.67, *P* < 0.001), and we found a significant difference between the nest and the random habitat characteristics (PERMANOVA: *F*_1,65_ *=* 2.27, *P* = 0.007) suggesting that females were selecting specific components of the available habitat. The interaction between habitat type and habitat plot type was not significant (PERMANOVA: *F*_1,65_ *=* 0.73, *P* = 0.81).

The habitat characteristics of the nest and territory plots in the unaltered and harvested habitat overlapped (Fig 3C). We found no difference in the centroids of the habitat space used by males compared to females (PERMANOVA: *F*_1,71_ *=* 1.51, *P =* 0.08) and no significant interaction between sex and forest habitat type (PERMANOVA: *F*_1,71_ *=* 0.88, *P =* 0.56). However, we found a significant difference in the centroids of habitat space by habitat type (PERMANOVA: *F*_1,71_ *=* 5.13, *P =* 0.002). To determine if the position of nest and territory plots occupied distinct regions in the habitat space, we used PERMANOVA to test for differences between the centroids of the territory space and in nest space between habitats in separate analyses. The habitat characteristics of male territories in the unaltered and harvested habitat overlapped; however, we found a significant difference between the centroids of this habitat space (PERMANOVA: *F*_1,26_ *=* 2.03, *P =* 0.01). There was also a significant difference between the centroids of the habitat space used for nesting in the unaltered compared to the harvested habitat (Fig 2; PERMANOVA: *F*_1,45_ *=* 3.997, *P <* 0.001).

### Is there evidence of a reduction or expansion of habitat space?

We did not find a significant difference in habitat heterogeneity, the amount of variation in habitat space, among forest habitat type (homogeneity of dispersions test, *F*_2,101_ = 2.7, *P* = 0.06). We used Tukey’s HSD to test for pairwise differences between groups and found no significant difference in dispersion (volume of the habitat space, as suggested by the ellipse sizes of the three sites in Fig 2) between the unaltered and burn habitats (*P* = 0.06), and between the harvested and the burn habitat (*P* = 0.09), and no difference in the volume of habitat space between the unaltered and the harvested habitats (*P* = 0.98). We found no difference in dispersion by habitat plot type (nest, territory, random) when all three stands were considered (*F*_2,101_ = 0.85, *P* = 0.44).

We did not find a difference in the volume of habitat space (e.g., niche breadth) occupied for nesting between the unaltered and selective harvest habitats (homogeneity of dispersion: *F*_1,45_ *=* 0.08, *P* = 0.77), and no difference in the volume of habitat space used by males in the two stands (homogeneity of dispersion: *F*_1,26_ = 0.34, *P* = 0.57). This indicates that the size of the habitat space used by males (territory) or by females for nesting did not differ in relation to forest habitat type.

### The Grinnellian niche: Are differences in habitat space related to reproductive success?

**Fig 4:**
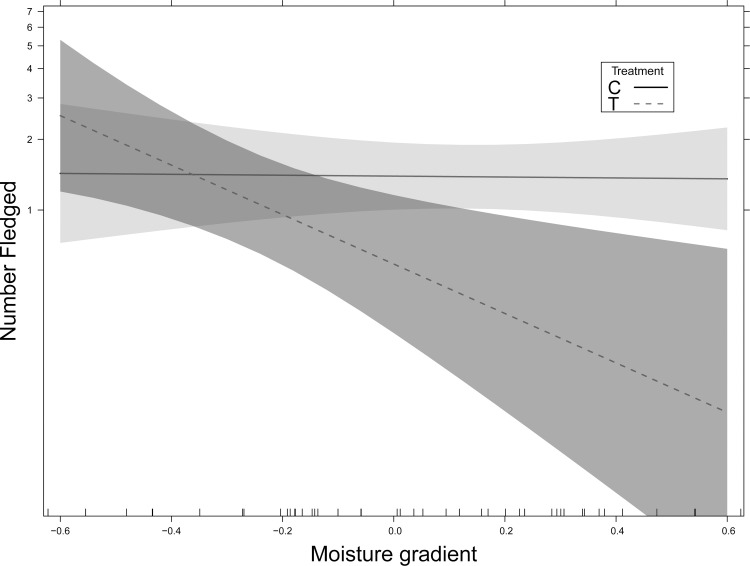
The relationship between number of fledged young and the interaction of forest habitat type and NMDS axis 1. Unaltered habitat = solid line; selective harvest habitat = dashed line with 95% confidence intervals.

We determined whether habitat structure explained variance in the number of young fledged per nest. Fewer young fledged in the selective harvest compared to the unaltered habitat (*z* = -2.45, *P* = 0.02). The AICc selected model (AIC weight: *w*_*i*_ = 0.13) explained 11% of the deviance in the number of fledged young and included forest type, NMDS1, the interaction between NMDS2 and NMDS1 and the interaction between forest habitat type and NMDS1. The number of young fledged was unrelated to the structural habitat characteristics in the unaltered habitat. However, in the harvested habitat more young fledged from nests in mesic habitat characterized by *Q*. *alba* and *Rubus* spp. than on xeric ridge tops (interaction of forest habitat type and NMDS 1: *z* = -2.41, *P* = 0.016, Fig 4). The second best model (*w*_*i*_ = 0.12) was consistent with the selected model but did not include the interaction between NMDS axis 1 and 2. All other models had low support (*w*_*i*_ < 0.06).

## Discussion

Within a population of hooded warblers, we detected differences in the habitat space occupied in relation to habitat characteristics in structurally different forest habitats. We found no difference in the size (volume) of habitat space used by males (territory) or by females (nesting); however there were differences among the centroids of habitat space in the three habitats. The centroids of the habitat space used by males and females in the unaltered and harvested habitats compared to the available habitat also suggest differences in the habitat characteristics selected by males and females. The habitat characteristics at nest locations in the harvested habitat were characterized by canopy white oaks with increased complexity in the shrub layer (increased Q. *alba* and *Q*. *rubra* saplings and *Rubus* spp.). In contrast, habitat characteristics at nests in the unaltered habitat were characterized by complex canopy structure with *Q*. *prinus* in the canopy and a shrub layer dominated by *Smilax* spp.. We also found that variation in habitat characteristics explained variation in the number of fledged young in the selective harvest but not the unaltered habitat. Consistent with our prediction, females in the harvested habitat occupied habitat space that was characterized by increased understory vegetation; however, the reduced fledging success indicates a potential reduction in niche space in this habitat. The ability to identify differences in habitat use and any corresponding differences in fitness measures can improve our understanding of how structurally different habitats affect demographic parameters within populations.

### Habitat space

Selective harvest and burning resulted in altered habitat structure compared to the unaltered habitat (S2 Fig) [40,41]. The burned habitat was similar to the unaltered habitat in canopy complexity and tree species composition but had reduced complexity in the understory. The habitat space in the harvested forest spanned a gradient from being dominated by canopy *Q*. *prinus* and *Smilax* spp. to space characterized by canopy *Q*. *alba* and *Rubus* spp. and overall had more complexity in the understory while the unaltered stand was characterized by a more complex canopy with *Q*. *prinus* and a shrub layer dominated by *Smilax* spp. (Fig 2, S1 Fig).

We found that the habitat used by female hooded warblers differed in relation to differences in habitat structure. In the harvested habitat, female nest sites were located in habitat space with increased understory complexity, using a subset of the available (random) habitat space (Fig 3B) while the habitat that the males used did not differ from the available habitat (Fig 3A). Males and females can exhibit different preferences for habitat structure [27,28,30] and hooded warblers exhibit habitat segregation on the wintering grounds [26,31]. For reproduction, males and females must occupy the same habitats on the breeding grounds but they may still exhibit different preferences in habitat structure and use different cues in selecting habitat [28]. Because of differences in morphology [50,51], foraging [29] or behavior [27,52], males and females may require different structural components and may have different adaptive peaks in relation to habitat use [26,27,31]. The habitat space used by females in the unaltered stand was similar to the available habitat; however, the habitat space used by females in both stands differed from the available habitat space and female nest site characteristics in the harvested habitat differed compared to the habitat characteristics used by females in the unaltered forest (Fig 3C). In contrast, males occupied habitat space that was more similar to the available habitat. Identification of sex differences in habitat space use and how habitat space affects reproductive success and survival will aid in our understanding of differences in abundance, density, and demography within or among habitats.

### The Grinnellian niche space differed among forest stands

We found a similar number of male territories in both the selective harvest and unaltered habitat (e.g., 12 territories in each stand during 2011 breeding season); however, we found fewer nests in the harvested habitat and fewer young were fledged per nest in the harvested habitat in both years of this study. In addition, habitat space in the harvested stand characterized by mesic slopes and more complex understory vegetation was associated with greater reproductive success than along the ridgetops in this stand. This suggests that the Grinnellian niche space (i.e., the habitat characteristics needed for survival and reproduction) differed and was reduced in the selective harvest stand. While the harvested habitat provided increased complexity in the understory, the reduced complexity in the canopy may have contributed to reduced reproductive success by providing more open perches for avian nest predators and avian nest parasites (i.e., brown-headed cowbirds, *Molothrus ater*) [53,54]. The reduced canopy complexity may have contributed to lower reproductive success in the harvested stand on the xeric ridge tops; while the greater understory vegetation complexity on the mesic slopes may have ameliorated the effects of avian predation and parasitism.

Burning reduced the Grinnellian niche space of hooded warblers in our burned study site. Male hooded warblers that occupied territories in the burn habitat primarily foraged, sang and displayed. We detected additional males singing or foraging in the burn; however, these individuals flew to adjacent non-burned habitat so were not included in analyses. One female each year nested in the burn habitat (the first year in a ravine characterized by *L*. *tulipifera*, *Plantus occidentalis* (sycamore) and *L*. *benzoin* that was unburned), but both nests failed because of predation during egg laying; therefore, the Grinnellian niche space for the hooded warbler was absent in the burn two years post burn. We occasionally observed other females in the burn habitat early in the breeding season but not by late May. In other southeastern Ohio forests, hooded warbler and ovenbird (*Seiurus aurocapilla*) either declined in burned habitat, using only portions of the habitat that were unaffected by burning for nesting, or they abandoned the habitat all together [55]. Although burned forests may provide habitat structure for foraging hooded warblers, there is a diminishment of the Grinnellian niche space in these stands due to lack of sufficient nesting substrate.

Differences in habitat structure can also induce behavioral trade-offs [56]. The foraging behavior of insectivorous birds can be affected by the thermal environment which alters insect community composition, distribution and activity level [57] which may in turn influence avian time and energy budgets. Indeed, biotic factors including caterpillar biomass, nest predation, double brooding and renesting explained 87% of the variation in fecundity of black-throated blue warblers [58]. In comparison, our best model linking the structural habitat characteristics to the number of fledged young only explained 11% of the variation. Predators and nest parasites do not compete for the vegetation component of the Grinnellian niche, rather habitat structure can affect a species’ ability to avoid predation and parasitism. Because predation is associated with variation in fecundity in Passerine birds [58,59] and predators can alter the behavior and nest placement of breeding birds [53,60], understanding what structural habitat characteristic are associated with variation in reproductive success can help us identify habitat space that may be associated with source and sink populations. Testing predictions about source-sink dynamics within a habitat mosaic can be accomplished by the addition of the above data to ecological niche models.

### The Grinnellian niche and conservation biology

Understanding how variation in habitat characteristics affect reproductive success and survival provides insight into variation in demographic data within or between populations. Because habitats used for breeding must provide the biotic and abiotic conditions for both survival and reproduction, it is important to consider multiple niche axes and determine if males and females require different characteristics within the niche space. Identification of factors that constitute niche space for populations across the landscape can be crucial for understanding population dynamics of target species. For example, Soberón and Nakamura [5] examined the environmental niche of the prickly pear moth (*Cactoblastis cactorum*), which is native to South America but has invaded Florida. They plotted the distribution of the moth in relation to temperature and precipitation and found the niche centroid in this space shifted in relationship to precipitation but not temperature. Similarly in the Sierra Nevada over the last 100 years, many of the bird species examined tracked a climate niche which resulted in shifted distributions [61]. The ability of individuals to track changes in biotic and abiotic conditions can promote population persistence in the face of land use and climate change

Identification of the structural characteristics that are important elements of the Grinnellian niche will aid in the identification and assessment of the factors that explain intraspecific niche variation and is another step toward understanding of the impacts of disturbance on populations as well as the distribution of species across a landscape. The availability of data on species presence/absence and detailed vegetation cover data allow for the assessment of broad scale patterns relating elements of the Grinnellian niche to species richness [4,14]. However, linking demographic data to the non-consumable resources used by a species and identifying combinations of resources that are associated with source sink dynamics can provide data to fine tune niche models in heterogeneous landscapes thus increasing our understanding of the impacts of habitat heterogeneity on the population dynamics.

## Supporting Information

S1 Fig Habitat space. NMDS of the habitat space based on 104 habitat plots from random, male territory center and female nest sites. Plant species and size classes as in S1 Table.
(PDF)

S2 Fig Random habitat space in the burned, harvested and unaltered habitat. NMDS of the random habitat space by habitat type.
(TIFF)

S1 Table Acronyms for the plant species sampled in the habitat plots, scientific name, common name, size class. Stem size classes: V1 < 3 cm, T1 = 10–23 cm, T2 = 23–38 cm, T3 > 38 cm and NMDS scores.
(PDF)
