# Supplementary figures and images for: Demographic Costs Associated with Differences in Habitat Space Occupancy
Source: PLoS One. 2016 Nov 16;11(11):e0165472. doi: 10.1371/journal.pone.0165472 (PMC5112845; doi:10.1371/journal.pone.0165472)

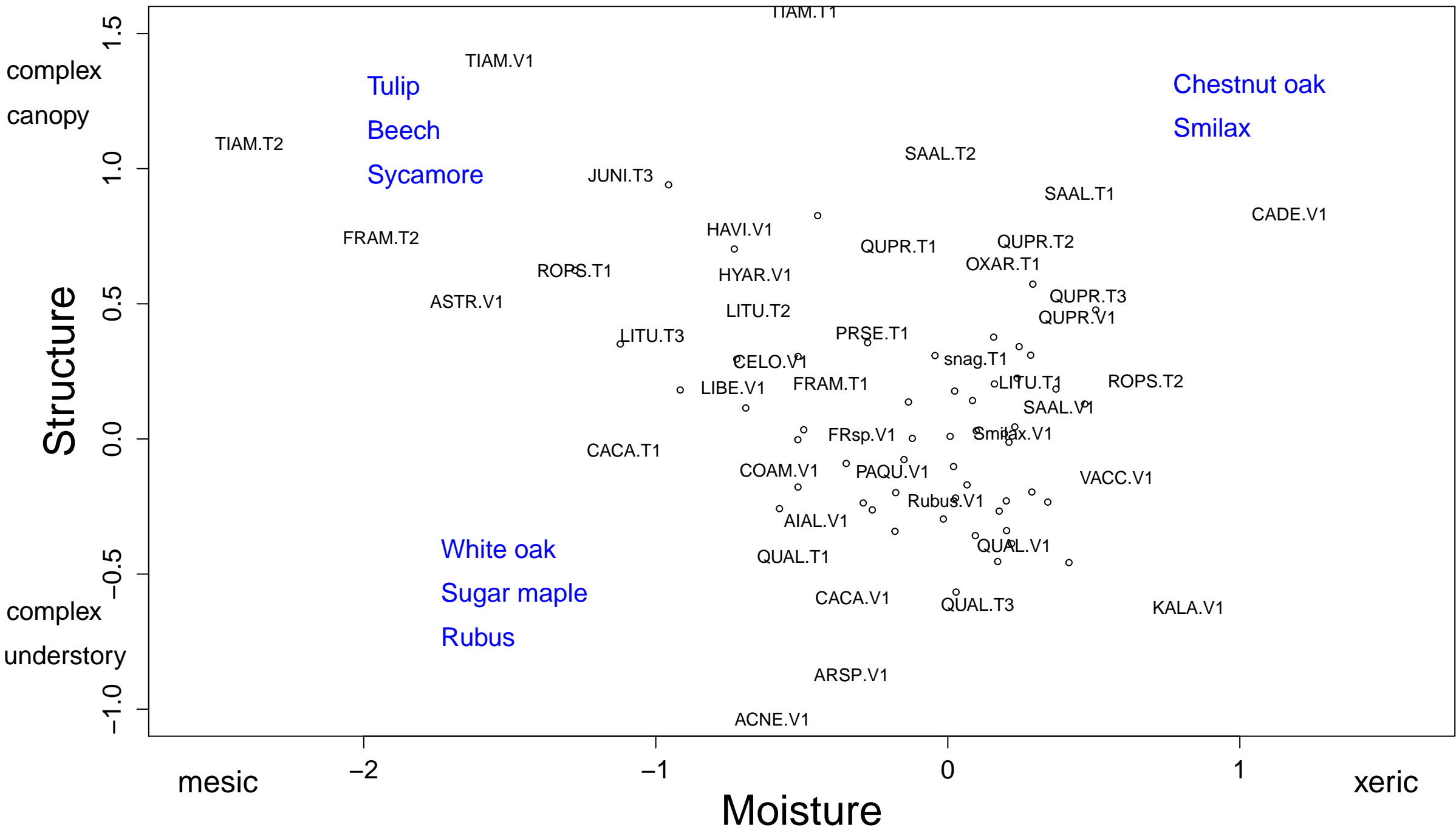

Supplement: S1 Fig — NMDS of the habitat space based on 104 habitat plots from random, male territory center and female nest sites. Plant species and size classes as in S1 Table. (PDF) [file pone.0165472.s001.pdf]

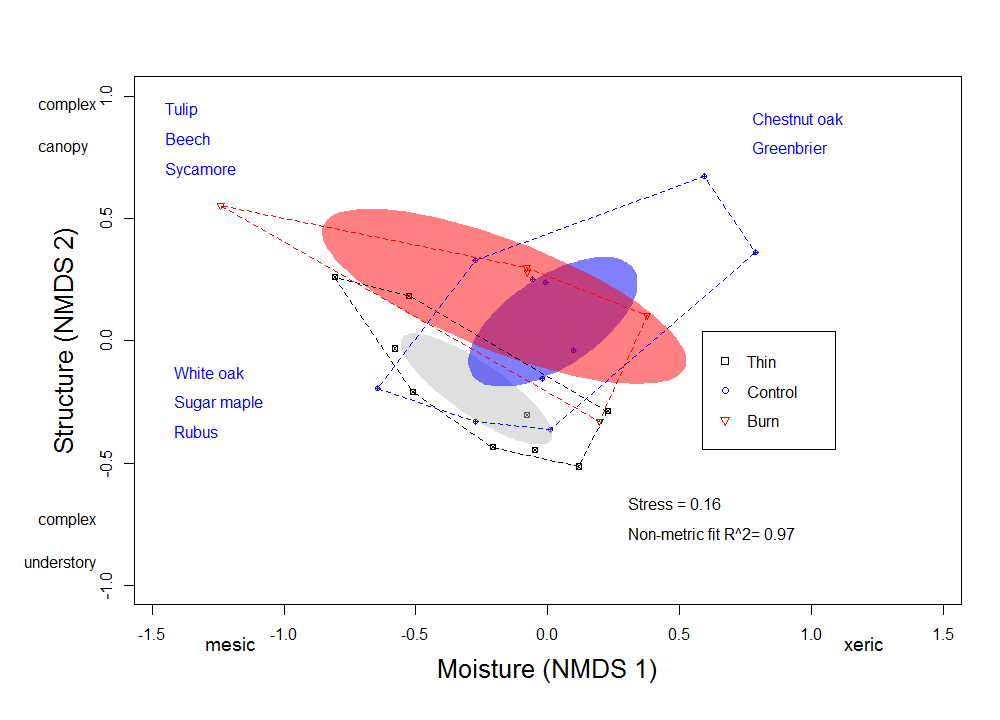

Supplement: S2 Fig — NMDS of the random habitat space by habitat type. (TIFF) [file pone.0165472.s002.tiff]
