# Supplementary material for: Demographic Costs Associated with Differences in Habitat Space Occupancy
Source: PLoS One. 2016 Nov 16;11(11):e0165472. doi: 10.1371/journal.pone.0165472 (PMC5112845; doi:10.1371/journal.pone.0165472)
Supplement: S1 Table — Stem size classes: V1 < 3 cm, T1 = 10–23 cm, T2 = 23–38 cm, T3 > 38 cm and NMDS scores. (PDF) [file pone.0165472.s003.pdf]

S1 Table. Plant species acronym, scientific name, common name, size class (Stem size classes: V1 < 3 cm, T1 = 10 – 23 cm, T2 = 23 – 38 cm, T3 > 38 cm) and NMDS scores.

| Code | Latin name | Common name | Size class | NMDS1 | NMDS2 |
|---|---|---|---|---|---|
| ACNE | *Acer negundo* | Boxelder | ACNE.V1 | -0.09 | -0.16 |
| ACRU | *Acer rubrum* | Red maple | ACRU.T1 | 0.44 | 0.30 |
| | | | ACRU.T2 | 0.25 | 0.37 |
| | | | ACRU.T3 | 0.18 | 0.03 |
| | | | ACRU.V1 | 0.24 | -0.28 |
| ACSA | *Acer saccharum* | Sugar maple | ACSA.T1 | -0.38 | -0.28 |
| | | | ACSA.T2 | -0.28 | -0.02 |
| | | | ACSA.T3 | -0.28 | 0.05 |
| | | | ACSA.V1 | -0.20 | -0.21 |
| AIAL | *Ailanthus altissima* | Tree of Heaven | AIAL.T1 | -0.10 | -0.03 |
| | | | AIAL.V1 | -0.28 | -0.04 |
| AMAR | *Amelanchier arborea* | Serviceberry | AMAR.V1 | 0.10 | -0.28 |
| ARSP | *Aralia spinosa* | Devil's walking stick | ARSP.V1 | -0.08 | -0.22 |
| ASTR | *Asimina triloba* | Pawpaw | ASTR.V1 | -0.36 | 0.15 |
| CACA | *Carpinus caroliniana* | Musclewood | CACA.T1 | -0.15 | 0.00 |
| | | | CACA.V1 | -0.14 | -0.28 |
| CADE | *Castanea dentata* | American chestnut | CADE.V1 | 0.22 | 0.12 |
| CARY | *Carya* spp. | Hickory spp. | CARY.T1 | -0.01 | -0.17 |
| | | | CARY.T2 | -0.11 | 0.06 |

| Code | Latin name | Common name | Size class | NMDS1 | NMDS2 |
|---|---|---|---|---|---|
| | | | CARY.T3 | 0.11 | -0.08 |
| | | | CARY.V1 | 0.07 | -0.22 |
| CELO | *Celtis Occidentalis* | Hackberry | CELO.V1 | -0.19 | 0.22 |
| CEOC | *Cercis canadensis* | Red-bud | CEOC.T1 | -0.18 | 0.18 |
| | | | CEOC.V1 | -0.37 | 0.17 |
| CLLU | *Cladrastis lutea* | Yellowwood | CLLU.V1 | -0.07 | -0.18 |
| COAM | *Corylus americana* | American hazel-nut | COAM.V1 | -0.15 | -0.11 |
| COFL | *Corus florida* | Flowering dogwood | COFL.T1 | -0.05 | 0.07 |
| | | | COFL.V1 | -0.08 | -0.10 |
| CRsp | *Crataegus spp* | Hawthorn | CRsp.V1 | -0.11 | -0.07 |
| FAGR | *Fagus grandifolia* | American Beech | FAGR.T1 | 0.08 | 0.14 |
| | | | FAGR.T2 | -0.09 | 0.03 |
| | | | FAGR.T3 | -0.17 | 0.04 |
| | | | FAGR.V1 | 0.00 | -0.05 |
| FRAM | *Fraxinus americana* | White ash | FRAM.T1 | -0.16 | 0.03 |
| | | | FRAM.T2 | -0.37 | 0.17 |
| | | | FRsp.V1 | -0.28 | 0.11 |
| HAVI | *Hamamelis virginiana* | Witchhazel | HAVI.T1 | -0.16 | 0.07 |
| | | | HAVI.V1 | -0.17 | 0.11 |
| HYAR | *Hydrangea arborescens* | Wild hydrangea | HYAR.V1 | -0.27 | 0.26 |
| JUNI | *Juglans nigra* | Black Walnut | JUNI.T3 | -0.34 | 0.32 |
| KALA | *Kalmia latifolia* | Mountain Laurel | KALA.V1 | 0.19 | -0.13 |

| Code | Latin name | Common name | Size class | NMDS1 | NMDS2 |
|---|---|---|---|---|---|
| LIBE | *Lindera benzoin* | Spicebush | LIBE.V1 | -0.63 | 0.22 |
| LITU | *Liriodendron tulipifera* | Tulip | LITU.T1 | 0.16 | 0.12 |
| | | | LITU.T2 | -0.24 | 0.14 |
| | | | LITU.T3 | -0.43 | 0.25 |
| | | | LITU.V1 | 0.02 | -0.22 |
| MORA | *Morus reubra* | Red Mulberry | MORU.V1 | -0.11 | 0.21 |
| NYSY | *Nyssa sylvatica* | Black Gum | NYSY.T1 | 0.16 | 0.09 |
| | | | NYSY.T2 | 0.11 | 0.08 |
| | | | NYSY.T3 | 0.00 | 0.05 |
| | | | NYSY.V1 | 0.24 | 0.05 |
| OXAR | *Oxydendrum arboreum* | Sourwood | OXAR.T1 | 0.11 | 0.21 |
| | | | OXAR.T2 | 0.06 | -0.03 |
| | | | OXAR.V1 | 0.27 | 0.26 |
| PAQU | *Parthenocissus quinquefolia* | Virginia creeper | PAQU.V1 | -0.26 | -0.16 |
| PIST | *Pinus strobus* | E. White Pine | PIST.V1 | 0.08 | -0.21 |
| PLOC | *Platanus occidentalis* | Sycamore | PLOC.T1 | -0.17 | 0.04 |
| | | | PLOC.V1 | 0.04 | -0.20 |
| PRSE | *Prunus serotina* | Black cherry | PRSE.T1 | -0.04 | 0.08 |
| | | | PRSE.V1 | 0.05 | -0.32 |
| QUAL | *Quercus alba* | White oak | QUAL.T1 | -0.22 | -0.12 |
| | | | QUAL.T2 | 0.01 | -0.30 |

| Code | Latin name | Common name | Size class | NMDS1 | NMDS2 |
|---|---|---|---|---|---|
| | | | QUAL.T3 | 0.09 | -0.59 |
| | | | QUEL.T3 | -0.05 | -0.15 |
| QUPR | *Quercus prinus* | Chestnut oak | QUPR.T1 | -0.01 | 0.19 |
| | | | QUPR.T2 | 0.20 | 0.43 |
| | | | QUPR.T3 | 0.46 | 0.50 |
| | | | QUPR.V1 | 0.42 | 0.35 |
| QURU | *Quercus rubra* | Red oak | QURU.T1 | 0.08 | 0.08 |
| | | | QURU.T2 | 0.06 | 0.11 |
| | | | QURU.T3 | 0.11 | 0.01 |
| | | | QURU.V1 | 0.27 | -0.45 |
| RHCO | *Rhus copallinum* | Winged sumac | RHCO.V1 | 0.03 | 0.08 |
| ROCA | *Rosa Carolina* | Carolina rose | ROCA.V1 | 0.05 | -0.20 |
| ROMA | *Rosa Multiflora* | Multiflora rose | ROMA.V1 | -0.26 | 0.03 |
| ROPS | *Robinia pseudoacacia* | Black locust | ROPS.T1 | -0.16 | 0.07 |
| | | | ROPS.T2 | 0.10 | 0.03 |
| | | | ROPS.V1 | 0.12 | -0.04 |
| Rubus | *Rubus* spp. | Blackberry spp. | Rubus.V1 | -0.01 | -0.29 |
| SAAL | *Sassafras albidum* | Sassafrass | SAAL.T1 | 0.10 | 0.26 |
| | | | SAAL.T2 | -0.02 | 0.30 |
| | | | SAAL.T3 | -0.12 | 0.13 |
| | | | SAAL.V1 | 0.34 | 0.23 |
| Smilax | *Smilax* spp. | Greenbrier | Smilax.V1 | 0.43 | 0.15 |

| Code | Latin name | Common name | Size class | NMDS1 | NMDS2 |
|---|---|---|---|---|---|
| Snag | na | snag | snag.T1 | 0.05 | 0.37 |
| | | | snag.T2 | -0.02 | 0.22 |
| | | | snag.T3 | -0.12 | -0.17 |
| TIAM | *Tilia americana* | Basswood | TIAM.T1 | -0.09 | 0.33 |
| | | | TIAM.T2 | -0.31 | 0.16 |
| | | | TIAM.V1 | -0.29 | 0.27 |
| TORA | *Toxicodendron radicans* | Poison Ivy | TORA.V1 | -0.25 | -0.23 |
| ULMU | *Ulmus* spp. | Elm spp. | ULMU.T1 | -0.11 | 0.05 |
| | | | ULMU.T2 | -0.04 | 0.26 |
